# Niche modeling predictions of the potential distribution of *Marmota himalayana*, the host animal of plague in Yushu County of Qinghai

**DOI:** 10.1186/s12889-016-2697-6

**Published:** 2016-02-24

**Authors:** Liang Lu, Zhoupeng Ren, Yujuan Yue, Xiaotao Yu, Shan Lu, Guichang Li, Hailong Li, Jianchun Wei, Jingli Liu, You Mu, Rong Hai, Yonghai Yang, Rongjie Wei, Biao Kan, Hu Wang, Jinfeng Wang, Zuyun Wang, Qiyong Liu, Jianguo Xu

**Affiliations:** State Key Laboratory of Infectious Disease Prevention and Control, Collaborative Innovation Center for Diagnosis and Treatment of Infectious Diseases, National Institute for Communicable Disease Control and Prevention, Chinese Center for Disease Control and Prevention, Beijing, 102206 China; State Key Laboratory of Resources and Environmental Information System, Institute of Geographic Science and Natural Resource Research, Chinese Academy of Sciences, Beijing, 100101 China; University of Chinese Academy of Sciences, Beijing, 100049 China; Key Laboratory of Surveillance and Early Warning on Infectious Disease, Chinese Center for Disease Control and Prevention, Beijing, 102206 China; Qinghai Institute for Endemic Disease Prevention and Control, Qinghai, 811602 China

**Keywords:** *Marmota himalayana*, Niche modeling, LST, GDEM, NDVI, Soil type

## Abstract

**Background:**

After the earthquake on 14, April 2010 at Yushu in China, a plague epidemic hosted by Himalayan marmot (*Marmota himalayana*) became a major public health concern during the reconstruction period. A rapid assessment of the distribution of Himalayan marmot in the area was urgent. The aims of this study were to analyze the relationship between environmental factors and the distribution of burrow systems of the marmot and to predict the distribution of marmots.

**Methods:**

Two types of marmot burrows (hibernation and temporary) in Yushu County were investigated from June to September in 2011. The location of every burrow was recorded with a global positioning system receiver. An ecological niche model was used to determine the relationship between the burrow occurrence data and environmental variables, such as land surface temperature (LST) in winter and summer, normalized difference vegetation index (NDVI) in winter and summer, elevation, and soil type. The predictive accuracies of the models were assessed by the area under the curve of the receiving operator curve.

**Results:**

The models for hibernation and temporary burrows both performed well. The contribution orders of the variables were LST in winter and soil type, NDVI in winter and elevation for the hibernation burrow model, and LST in summer, NDVI in summer, soil type and elevation in the temporary burrow model. There were non-linear relationships between the probability of burrow presence and LST, NDVI and elevation. LST of 14 and 23 °C, NDVI of 0.22 and 0.60, and 4100 m were inflection points. A substantially higher probability of burrow presence was observed in swamp soil and dark felty soil than in other soil types. The potential area for hibernation burrows was 5696 km^2^ (37.7 % of Yushu County), and the area for temporary burrows was 7711 km^2^ (51.0 % of Yushu County).

**Conclusions:**

The results suggested that marmots preferred warm areas with relatively low altitudes and good vegetation conditions in Yushu County. Based on these results, the present research is useful in understanding the niche selection and distribution pattern of marmots in this region.

## Background

Plague, caused by the gram-negative bacterium *Yersinia pestis*, is a serious infectious disease and anthropozoonosis, which occurs in natural foci and is mainly hosted by rodents [1, 2]. Plague has given rise to at least three major pandemics, and the third originated in southwest China [2]. The highest diversity and largest area of plague foci are in China, which are hosted by different groups of rodents, including the ground squirrel, marmot, gerbil, vole and rat [3]. Therefore, continuous surveillance of the density of rodents and the infection rate of *Y. pestis* in rodent populations is important for the prevention of plague epidemics in China.

There are several limitations with current plague surveillance programs for monitoring infection in rodent hosts. The present monitoring program is time-consuming, labor intensive and costly, and can not detect emerging zoonotic sites easily, especially in foci hosted by marmots and distributed in high altitude areas. For example, every year, a monitoring site in a marmot plague focus of China should survey the density of marmots twice, the density of vector fleas and nocturnal rodents every month, and check more than 100 marmot samples with serologic and pathogenic methods in an area of more than 200 km^2^ during the active season of marmots [4]. Therefore, monitoring host populations with modern techniques that can give more data about the host distribution and density in large scale areas would be useful.

The comprehensive application of the geographical information system (GIS), remote sensing (RS), and global positioning system (GPS) showed strong potential in public health research and surveillance [5] and has been widely used in the monitoring, prediction and control of infectious diseases. In plague monitoring in Kazakhstan, satellite images were used to locate new or expanding foci without field works, measuring the density of great gerbil burrow systems, and to perform direct surveillance and control efforts [6]. The same research was also conducted at the great gerbil focus at Xingjiang and the marmot focus at Gansu in China [7, 8], and the results could be used in predicting the distribution of host animals without further field works. GIS models, such as the ecological niche model (ENM), neural network model and Bayesian model, have been widely applied in infectious disease prediction [9, 10]. ENM, as a present-only model, has achieved good results in disease analysis and disease prediction [8].

The earthquake that occurred on 14 April 2010 at Yushu in China caused severe loss of human life and economy. At the beginning of the rescue, the public health authority noticed that Yushu county was an active area of plague zooepidemic hosted by Himalayan marmot (*Marmota himalayana*), and after marmots waking up from hibernation in May there would be high risk of plague epidemic, especially for the rescue teams from other provinces [11]. This kind of risk would be higher in the reconstruction period in following years because there would be more than ten thousands builders lived and worked dispersedly in the area at where marmots lived. Thus, a risk assessment of the plague caused by the Himalayan marmot in different areas of Yushu was necessary for the reconstruction and development of the city.

Although there was a plague surveillance team at Yushu, the surveillance data from one surveillance site could not be used to deduce the the distribution and density of marmot in other areas. And the investigation of the Himalayan marmot in an area of 15,116 km^2^ was an impossible mission for the surveillance team. If the habitat fit for the Himalayan marmot could be defined with an easy and adaptable method, a rapid risk assessment of the total area would be possible.

In this research, Yushu County in Qinghai, which is located in the natural focus of the Himalayan marmot plague, was chosen as the study area. The positions of the burrow systems of the Himalayan marmot were investigated and recorded with GPS. The environmental factors that may affect the spatial distribution of the burrow system were determined. Based on these data, the relationship between the environmental factors and the distribution of burrow systems was analyzed to predict the geographic distributions of the burrows of marmots, including the distribution and the density of marmots in this area.

## Methods

### Study area

**Fig. 1:**
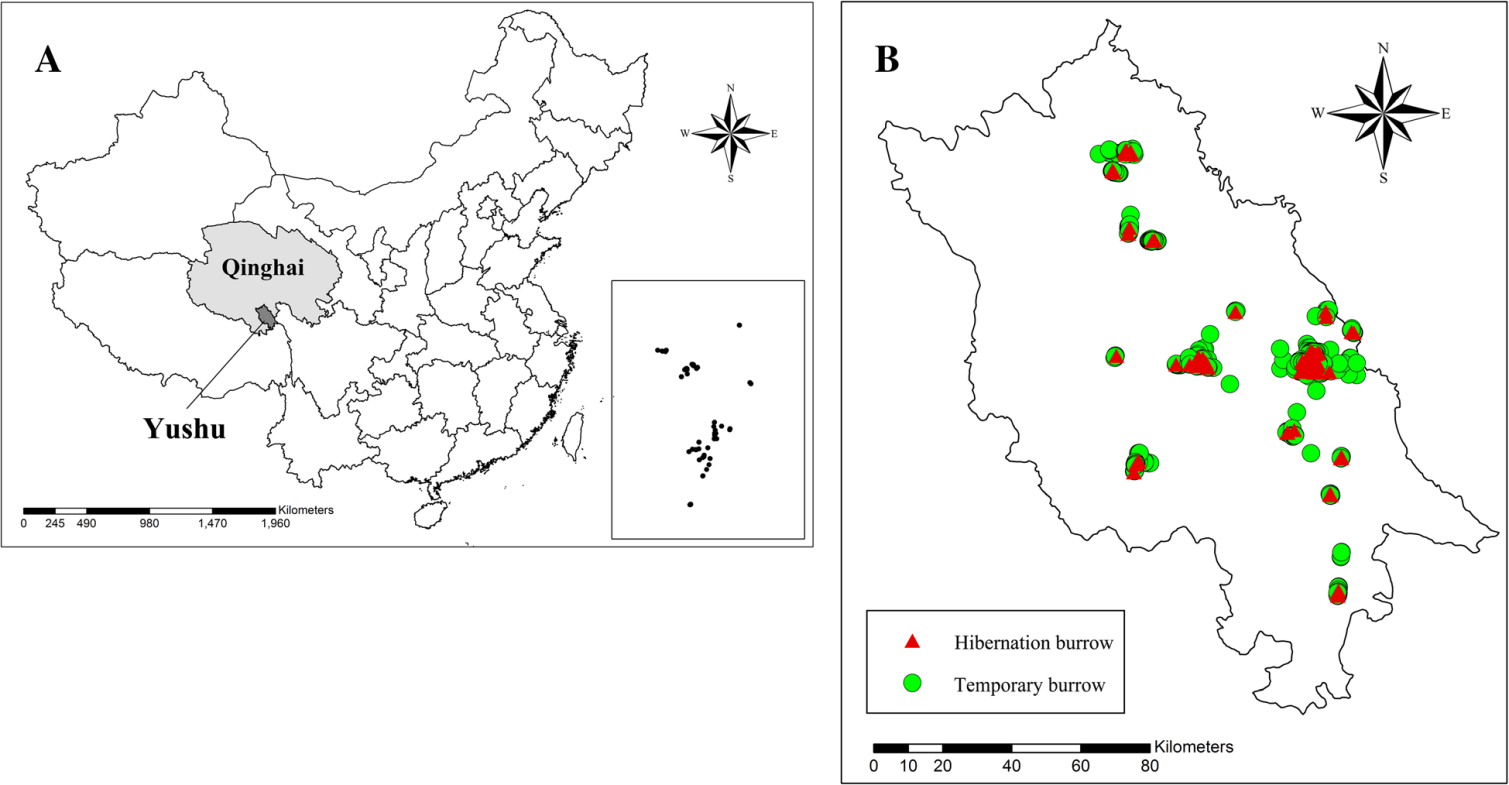
The location of research areas and the distribution of the investigated burrows. **a** The location of Yushu County in Qinhai of China; **b** The distribution of investigated hibernation (*red*) and temporary (*green*) burrows of marmot in Yushu County

Yushu County is located in the central region of the Tibetan Plateau, at the south of the Bayan Har Mountain and the Yangtze River’s headwater area. The county area is 15,116 km^2^ (Fig. 1a). The area has an average elevation of 4506 m (Table 1), and the terrain is complex and towering. Yushu has a classic alpine subarctic climate, with a long winter and short summer. Moreover, it is very cold and dry in the winter and rainy and mild in the summer. June is the rainiest month.

**Table 1:** Descriptive statistics of environmental variables used in Maxent ecological niche modeling of burrow site selection by Himalayan marmots. The summer value of LST and NDVI are averages of 3-year values from May 9th to September 30th, and the winter values are averages of 3-year values from 3rd December and 5th March. The meanings of abbreviations are listed in legend of Fig. 2

Variables	Minimum	Maximum	Mean
LST_summer	6.2	27.8	19.1
LST_winter	−10.0	16.4	6.7
NDVI_summer	0.01	0.71	0.52
NDVI_winter	−0.14	0.58	0.19
Elevation	3400	5584	4506
Soil type	1. Grey cinnamon soil, 2. Swamp soil, 3. Alpine meadow soil, 4. Dark felty soil, and 5. Frigid soil.		

### Data

Data in this study included in situ sampling and environmental factors. The environmental factors included the land surface temperature (LST) in winter and summer, normalized difference vegetation index (NDVI) in winter and summer both derived from the Moderate resolution Imaging Spectroradiometer (MODIS) satellite, Advanced Spaceborne Thermal Emission and Reflection Radiometer (ASTER) and Global Digital Elevation Model (GDEM), and soil type data. All data adopted in this area were resampled to 1 km spatial resolution.

### In situ investigation

The Himalayan marmot lives in family units which may join together to form colonies, the size of which depends on the resources available [12]. In some cases, a colony can contain up to 30 families [13]. The density of himalayan marmot, which was always surveyed with observation method, ranged from 20 to 130 individuals per km^2^ at east and south of Qinhai province. A Himalayan marmot family usually has 5–8 individuals, includes one couple of adults, infants and juveniles with 0–2 years old [14].

In the burrow systems of Himalayan marmot, the hibernation burrow was the place where members of family or close related families hibernated together during winter, and the temporary burrows were dug to avoid predators or just for fun, which could be shared among members of close related families. Some temporary burrows were interlinked with hibernation burrows underground. Because of the deep digging to create a large internal space, there was lots of gravel around the entrance of the hibernation burrows, which can be used to distinguish the two types of burrows. The number of hibernation burrows indicated the number of marmot families in an investigated area, and the number of temporary burrows reflected the number of individuals in the area.

During the active season of marmot, the localities of the hibernation burrows and temporary burrows of marmots were investigated from June to September in 2011. The field investigation was permitted and supported by National Institute for Communicable Disease Control and Prevention, China CDC, and Qinghai Institute for Endemic Disease Prevention and Control, and got administration permission from Yushu County Center for Disease Control and Prevention for the actives in the focus area of plague. Sampling quadrates were selected along valleys with stable direction. The long of quadrates was about 250 m along the valley, the wide of quadrates depended on the distribution of burrows on the slope, ranged from 100 to 200 m.

The location of every burrow was geo-located through a portable global positioning system (GPS). Figure 1b shows the distributions of the two types of burrows in Yushu County.

### MODIS LST and NDVI

MODIS is a key instrument aboard the Terra and Aqua satellites. 8-day composite MODIS LST and 16-day composite MODIS NDVI, both at a resolution of 1 km, were used to represent the thermal environment and feeding conditions for the Himalayan marmot, respectively. NDVI values vary between −1 and +1; the higher the NDVI value, the denser the green vegetation [15]. These two remote sensing variables were obtained from the MODIS website (https://lpdaac.usgs.gov/dataset_discovery/modis/modis_products_table). To characterize the thermal and vegetation conditions more reliably (reducing the uncertainty of the remote sensing data), we averaged the LST and NDVI values for three years prior to the survey time (that is 2009, 2010 and 2011). Eventually, we chose December 3rd to March 5th with the mean LST of 6.7 °C as the cold days and May 9th to September 30th with the mean LST of 19.1 °C as the warm days. Therefore, the mean NDVI during the cold days was 0.19, and the mean NDVI during the warm days was 0.52. LST_winter and LST_summer represented the LST during the cold days and LST during the warm days, respectively. NDVI_winter and NDVI_summer represented NDVI during the cold days and NDVI during the warm days, respectively (Table 1).

### ASTER GDEM

The ASTER GDEM product (Version 2) with 30 m spatial resolution was available from the NASA Land Processes Distributed Active Archive Center (https://lpdaac.usgs.gov/dataset_discovery/aster/aster_products_table/astgtm). The ASTER GDEM was compiled from over 1.5 million ASTER scenes acquired between March 2000 and August 2010. GDEM was based on ASTER stereoscopic data of nadir and backward-looking near-infrared channels 3A and 3B at 15 m spatial resolution. A newly developed cloud-mask function was applied to remove cloudy pixels. All cloud-screened GDEMs were stacked onto a global grid with a horizontal spatial resolution of 30 m, and a statistical selection algorithm was used to remove abnormal values and outliers. Enhanced accuracy was achieved by using multiple scenes from the same area (LPDAA) (https://lpdaac.usgs.gov/dataset_discovery/aster/aster_products_table). Most of the burrows in this study area occurred at an elevation of 3700–4700 meters.

### Soil type

Soil data with 1 km spatial resolution was available from the Nanjing Institute of Soil Science, Chinese Academy of Sciences (http://www.issas.ac.cn/). There were five types of soil in the study area, namely grey cinnamon soil, swamp soil, alpine meadow soil, dark felty soil and frigid soil (Table 1).

### Ecological niche modeling (Maxent)

Maxent is a machine learning model that uses presence-only data (occurrence records of burrows in this research) and environmental variables to build relationships based on the principle of maximum entropy [16]. The basic principle of the Maxent model is to estimate the potential distribution of a species by determining the distribution of the maximum entropy (i.e., closest to uniform), with constraints imposed by the observed spatial distributions of the species and the environmental conditions [16–18]. Maxent computes a probability distribution that describes the suitability of each grid cell (varying from 0 to 1, indicating the lowest suitability and the highest suitability, respectively) [19] as a function of the environmental variables at the known occurrence locations [20] and then produces a map of the species’ potential geographical distribution by projecting into the geographic space. Maxent is the best model for presence-only data according to the prediction accuracy across several ecological niche modeling methods [21].

The Maxent software version 3.3.3 k (available from http://www.cs.princeton.edu /~schapire/maxent/) was adopted in this study to predict the potential distribution of hibernation and temporary burrows of the Himalayan marmot. To evaluate the model performance, the data were split into two parts: 75 % for training and 25 % for test sets [18, 22]. A 10-fold cross-validation was used to perform the model training and testing to assess the performance of our model. The Jacknife test was used to determine the percentage contribution of each environmental variable to the predictive model. The test gain and test area under the receiving operator curve (AUC) were used to evaluate the model’s goodness-of-fit. The AUC was an effective indictor of model performance. The larger the AUC, the highest was the sensitivity rate, and the lower was the 1-specificity rate. In general AUC values of 0.5–0.7 were considered as low accuracy, values of 0.7-0.9 were considered as useful applications and values of >0.9 were considered as high accuracy [20]. The “10th percentile training presence logistic threshold” was adopted to convert the continuous presence probability maps into suitable and unsuitable areas [18, 19].

## Results

There were 124 hibernation burrows and 1738 temporary burrows in 51 sampling quadrates in the study area. Number of hibernation burrows in quadrates ranged from 0 to 11. Environmental factors, LST, NDVI, GDEM and soil types, were taken into account. The relationships of the hibernation/temporary burrows and environmental factors were analyzed. Furthermore, the potential distribution of the Himalayan marmot was explored.

### Model performance

Table 2 shows the model accuracy for hibernation/temporary burrows. The models for hibernation and temporary burrows both performed very well with average test AUC values of 0.819 and 0.809, respectively, indicating good discriminative ability between suitable and not suitable areas, and with average test gain values of 0.761 and 0.697, respectively, indicating passable goodness-of-fit. The models showed that the distribution of hibernation and temporary burrows was related to different environmental factors. The contribution orders of the variables in the models were LST_winter, Soil type, NDVI_winter, GDEM, and LST_summer, NDVI_summer, Soil type, and GDEM. The distribution of the suitable area for hibernation burrows was largely affected by LST_winter, whereas the distribution of the suitable area for temporary burrows was mainly controlled by LST_summer. For hibernation burrows, LST_winter was the most important predictor (percent contribution: 78.4 %); soil type was next (percent contribution: 11.6 %) but was substantial lower than LST_winter. Although LST_summer was also most important for the distribution of temporary burrows (percent contribution: 53.7 %), NDVI_summer, with the exception of soil type, was the secondary important predictor for temporary burrows (percent contribution: 22.6 %). For both hibernation and temporary burrows, the model was built from LST and the occurrence localities of the burrows had the largest test AUC values (test AUC = 0.793 and 0.779, respectively) and test gain values (test gain: 0.608 and 0.520). The model with LST excluded performed significantly worse for the hibernation or temporary burrows. The model with NDVI_summer excluded performed significantly worse for the temporary burrows. For both the hibernation and temporary burrow models, LST, NDVI and GDEM indicated good discriminative ability, with test AUC values of approximately 0.7.

**Table 2:** The Maxent ecological niche modeling results for hibernation burrows and temporary burrows

Hibernation burrow (Test AUC: 0.819; Test gain: 0.761)					
Variable	Percent contribution	Test gain (Variable alone)	Test gain (Variable excluded)	Test AUC (Variable alone)	Test AUC (Variable excluded)
LST_winter	78.4	0.608	0.719	0.793	0.815
Soil type	11.6	0.239	0.719	0.681	0.818
NDVI_winter	5.5	0.378	0.736	0.712	0.815
GDEM	4.5	0.524	0.707	0.778	0.813
Temporary burrow (Test AUC: 0.809; Test gain: 0.697)					
Variable	Percent contribution	Test gain (Variable alone)	Test gain (Variable excluded)	Test AUC (Variable alone)	Test AUC (Variable excluded)
LST_summer	53.7	0.520	0.463	0.779	0.769
Soil type	4.7	0.159	0.668	0.639	0.806
NDVI_summer	22.6	0.259	0.610	0.673	0.792
GDEM	1.9	0.321	0.631	0.733	0.798

### Relationships between burrow occurrence and environmental variables

**Fig. 2:**
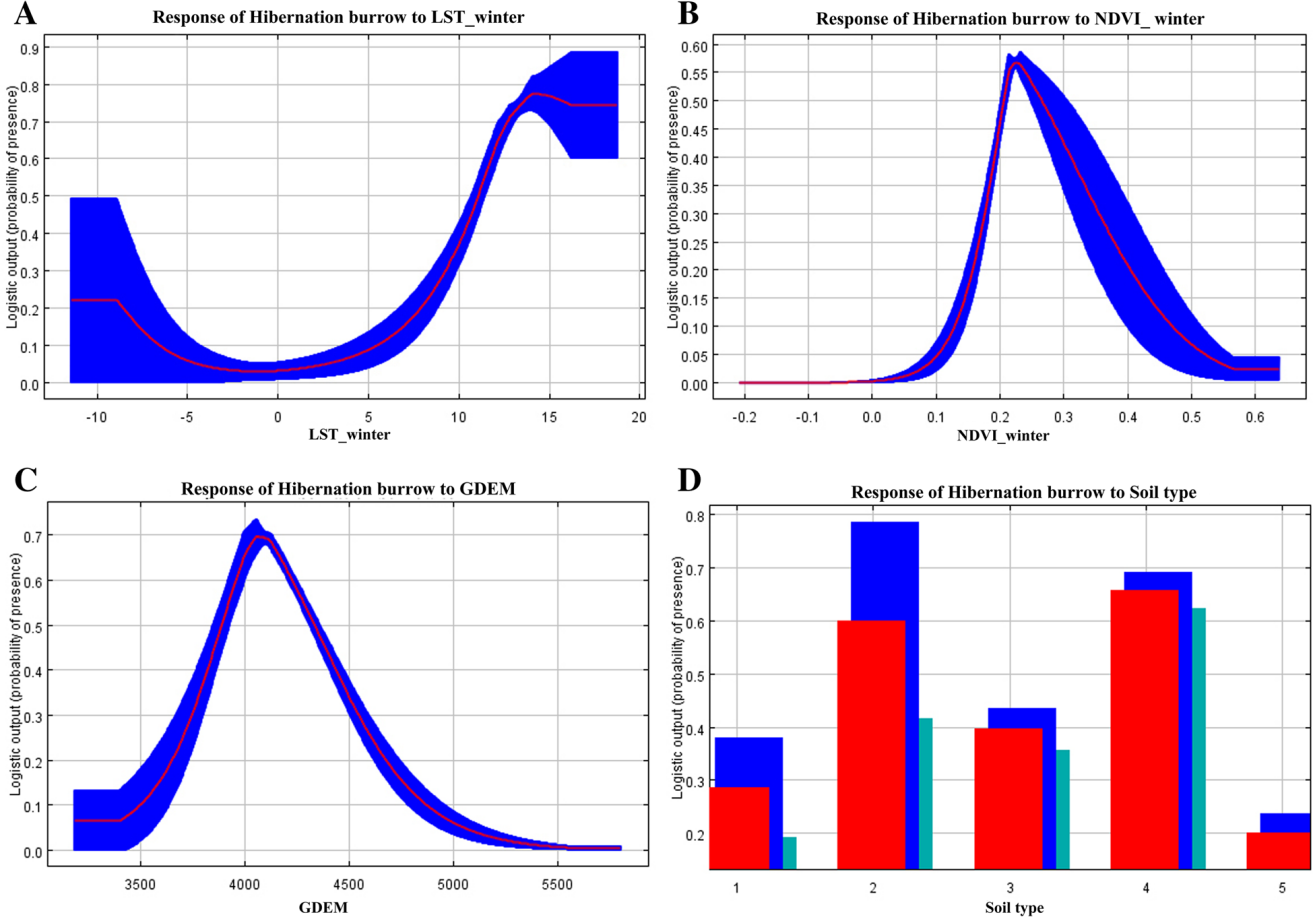
Response curves and data for each environmental variable in ecological niche modeling of hibernation burrows. **a** LST_winter: land surface temperature during the cold days; **b** NDVI_winter: normalized difference vegetation index during the cold days; **c** GDEM; **d** Soil type: 1 Grey cinnamon soil, 2 Swamp soil, 3 Alpine meadow soil, 4 Dark felty soil and 5 Frigid soil. The red curves (**a**, **b**, **c**) and the red bar (**d**) show the mean response of 10 replicate runs, the blue shadow (**a**, **b**, **c**) and the blue and green bars (**d**) show the mean +/− one standard deviation

**Fig. 3:**
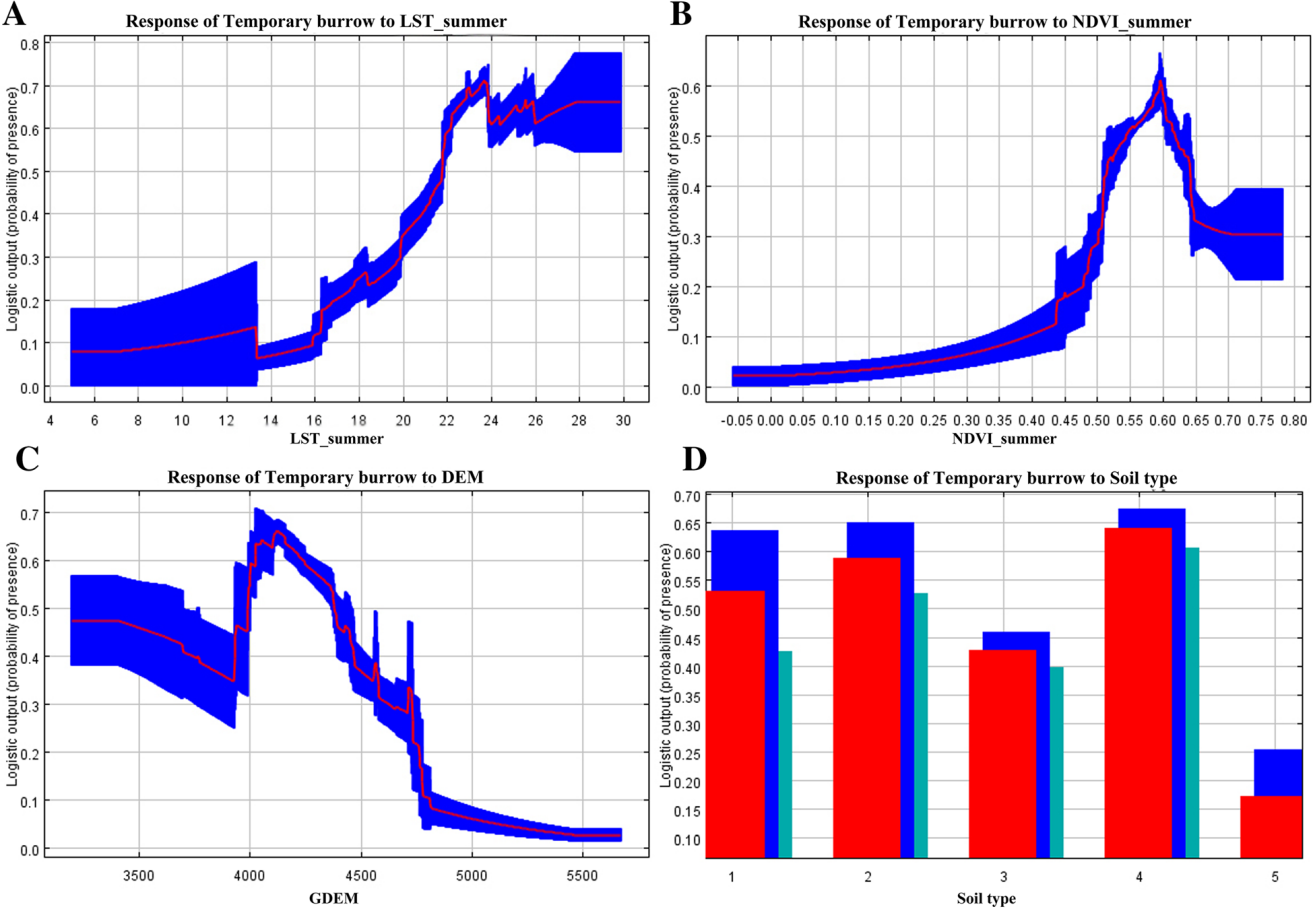
Response curves and data for each environmental variable in ecological niche modeling of temporary burrows. **a** LST_summer; **b** NDVI_summer; **c** GDEM; **d** Soil type: 1 Grey cinnamon soil, 2 Swamp soil, 3 Alpine meadow soil, 4 Dark felty soil and 5 Frigid soil. The red curves (**a**, **b**, **c**) and the red bar (**d**) show the mean response of 10 replicate runs, the blue shadow (**a**, **b**, **c**) and the blue and green bars (**d**) show the mean +/− one standard deviation. The meanings of abbreviations are listed in legend of Fig. 2

Figures 2 and 3 show the response curves between the environmental variables and the prediction changes of hibernation/temporary burrows, respectively. The relatively narrow confidence interval indicated that the models were robust. The response curve for hibernation burrows showed a clear non-linear relationship between the probability of hibernation burrow presence and LST_winter (Fig. 2). The probability of hibernation burrow presence decreased slightly when LST_winter was −10 °C to 0 °C, whereas the probability gradually increased when LST_winter ranged from 0 to 14 °C. However, there was a non-linear positive response observed between temporary burrow and LST_summer, which was greater than 6 °C, and the probability of temporary burrow presence decreased slightly when LST_summer was greater than 23 °C (Fig. 3). There was also non-linear relationship between the probability of burrow presence and NDVI. The probability of hibernation burrow presence peaked when NDVI_winter was 0.22 (Fig. 2). In contrast, the probability of temporary burrow presence peaked when NDVI_summer was 0.60 (Fig. 3). There was also a non-linear relationship between the probability of burrow presence and GDEM. The probability of hibernation/temporary burrow presence peaked when GDEM was 4100 m (Figs. 2 and 3). A substantially high probability of hibernation/temporary burrow presence was observed in soil types 2 (swamp soil) and 4 (dark felty soils) compared to the other soil types (Figs. 2 and 3).

### Prediction of suitable areas for hibernation and temporary burrows

**Fig. 4:**
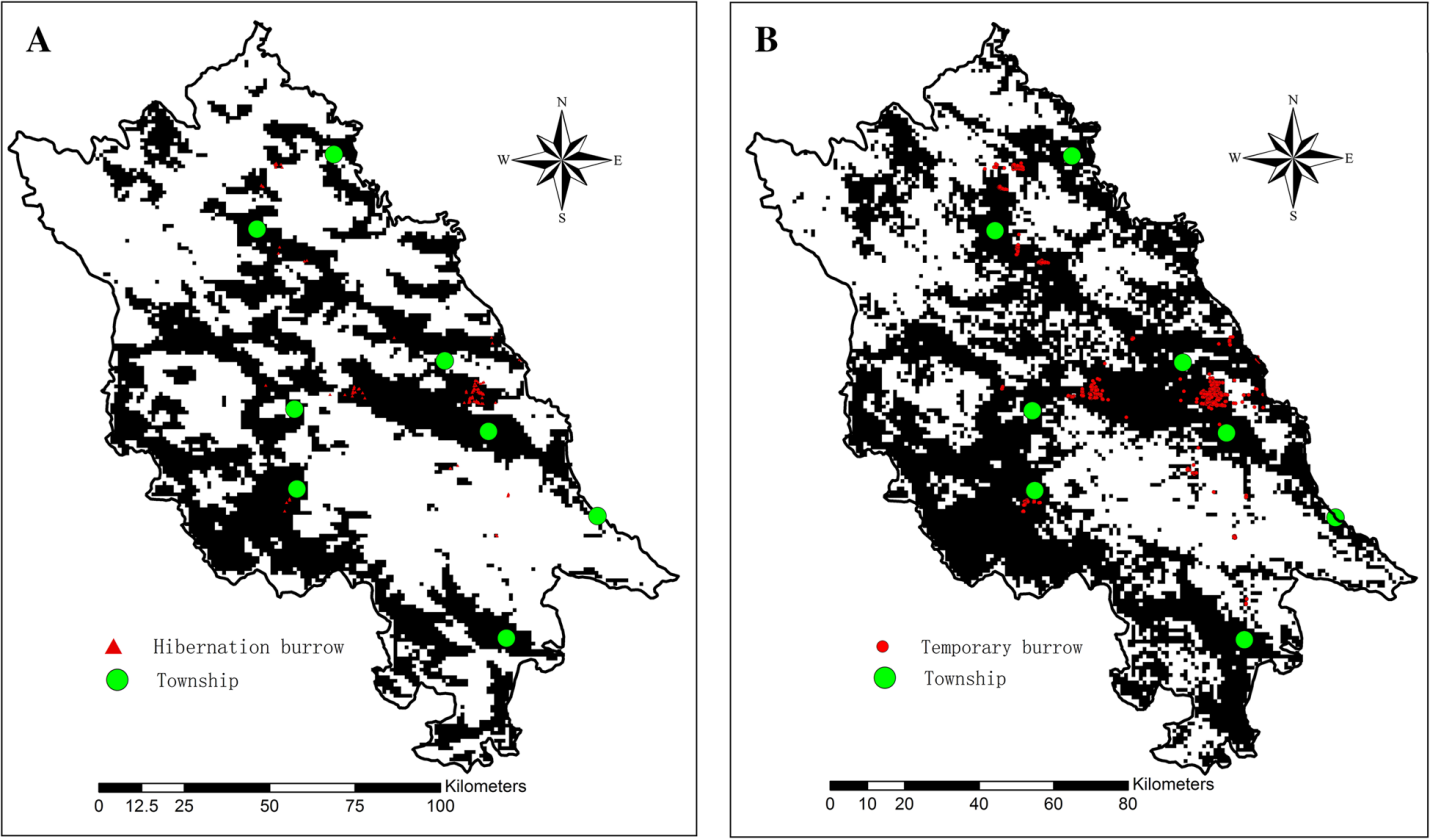
The potential suitable areas for hibernation and temporary burrows in Yushu County. **a** The suitable area (*black area*) for hibernation burrows, which was 5696 km^2^, the triangle red dots are the investigated hibernation burrows in present research. **b** The suitable area (*black area*) for temporary burrows, which was 7711 km^2^, the red dots is the investigated temporary burrows. The green dots show the locations of townships of Yushu county, which are all surrounded by the active areas of Himalayan marmot

Figure 4 shows the potential suitable areas for hibernation and temporary burrows in Yushu County. The predicted areas for hibernation and temporary burrows both covered a large area extending from north to south in the study area and showed homogeneity and heterogeneity between each other. According to the 10th percentile training presence logistic threshold, 0.203 was adopted for the prediction map of hibernation burrow presence, and 0.24 was adopted for the prediction map of temporary burrow presence. Finally, the potential suitable area for hibernation burrows was 5696 km^2^ (37.7 % of Yushu County area) and 7711 km^2^ (51.0 % of Yushu County area) for temporary burrows.

## Discussion

Some marmot species (*M. himalayana*, *M. sibirica*, *M. baibacina*, *M. caudate* and *M. flaviventris*) are important host animals of plague in northeast Asia and North America [3, 23, 24]; thus, the traits of the habitat and environmental factors determining the distribution of marmots have been analyzed in several studies. Anderson and Bopp studied the influence of facing direction and slope angle of the slope to marmot burrow distribution, and concluded that the slop angle was important to the distribution of burrow system [22, 25]. Svendsen studied the environmental factors controlling the distribution of the burrows of yellow-bellied marmots [26]. Armitage [27, 28] summarized the preferred habitats of marmots, including “1) meadow or grassland for foraging; 2) eastern to southern exposure where snow melts earlier than on northern or western exposures; 3) a moderate to steep slope that provides good drainage; 4) a solid structure that supports a burrowing habit and often associated with rocks or talus; and 5) typically at high elevations”. Four key factors determining the ecological niche of the Himalayan marmot have been identified in the Central Himalayas (Nepal), including elevation, temperature, the presence of accumulative formations and feeding conditions [13]. Similar results indicated that temperature, vegetation and elevation were important factors for the distribution of Himalayan marmots distributed in China [7, 8]. Based on these studies, four factors that can be retrieved from remote pictures (LST, NDVI, GDEM and soil type) were selected in the present research.

Environmental temperature is a key variable to control the geographical distribution of marmot burrows. Marmot physiology is highly adapted to coping with low temperature but is restricted by high temperature in the summer [27, 28]. The temperature value (22–25 °C) at which marmots suffer from heat stress is critical, and their activity on the surface sharply decreases [29]. However, the temperature data of remote areas are always unavailable from meteorological departments; thus, LST data from remote sensing pictures are the best choice to show the spatial change of the environmental temperature in such a large area. The results of the present research showed that the distribution of temporary burrows, which indicates the active area of the marmots, was positively related with the LST in summer when LST ranged from 14 to 24 °C, which was similar to the results of Türk and Arnold [29]. The relationship between the distribution of hibernation burrows and LST_winter suggested that marmots preferred warm areas for hibernation burrows. In these areas, the snow cover melts early in spring; thus, the survival and reproduction rate of the populations in these areas are higher than that of populations in other areas [28].

NDVI is an indicator of the feeding conditions of marmots. Feed plants are another important factor in marmot growth and survival [28]. Gao, Li et al. [7] reported that the number of marmot burrows is the largest when the grass height is between 10 and 15 cm and vegetation coverage is more than 90 % in the areas between 3200 and 3500 m. In the present research, most of the temporary burrows were distributed in areas with NDVI in summer ranging from 0.5 to 0.65. There are two main types of vegetation in Yushu County, alpine meadow at lower altitudes and shrub above the meadows. The shrub provides high NDVI values, but it is not the preferred habitation of marmots.

Armitage [28] noted that the soil type was also an important factor for the distribution of marmots. The best soil for marmots has special structure to support the large and complex burrow systems. The Himalayan marmot habitat in the Central Himalayas was found to abound in accumulative formations, such as alluvial, deluvial, and fluvioglacial deposits, which are of light texture, no less than 10 m deep, and ideal for burrowing [13]. In the present research, more burrows were found in swamp soil and dark felty soils, which meant that in Yushu County, soil type was also an important factor for the distribution of the Himalayan marmot.

In the present research, most marmot burrows were found in areas from 3800 m to 4400 m, especially near 4100 m. During the investigation, marmot burrows were found in river banks and valley floors with good vegetation conditions. In China, the Himalayan marmot is found at areas above 2500 m in the northern Qinghai and above 4500 m in northern Yunan. In Nepal, the Himalayan marmot is found in areas above 3000 m [30]. Therefore, marmots prefer the areas with good conditions rather than a specific elevation.

According to the prediction map of hibernation/temporary burrows, the potential area for hibernation burrows was 5696 km^2^ (37.7 % of Yushu County), and the potential area for temporary burrows was 7711 km^2^ (51.0 % of Yushu County). Therefore, the active area of the Himalayan marmot is broad. Most of the suitable areas for the Himalayan marmot are distributed in areas with low elevation and high LST, in which all townships are located (Fig. 4). The sympatric distribution of marmots and human being increases the dangers of infection and epidemic of plague, especially for construction teams and tourists. Therefore, sufficient health education for local residents, construction teams and tourists from other provinces is necessary to prevent infection and epidemic plague in these areas.

Comparing to the traditional field work methods, remote sensing could provide different types of data of a huge area at the same time. Processed with GIS software, these data could be used to evaluate the environmental characters of the investigated area for ecological and environmental purposes. In present paper, data collected from field investigation were successfully correlated with data from remote sensing, and provided the niche selection of Himalayan marmot in Yushu county. Based on these results, the distribution area of Himalayan marmot in 2011 was estimated. Whit the same method, the distribution of Himalayan marmot in following years could also be estimated with the environmental data collected with remote sensing. Furthermore, the density of marmot in the distribution area could also be calculated according the remote sensing data only after the relationships of marmot density and environmental factors were established based on further field work.

## Conclusions

The results of the present research showed that combined with data from field investigation, remote sensing data can be used to analyze and predict the distribution areas of the Himalayan marmot. In China, plague nature foci hosted by marmot, ground squirrel, vole and gerbil are located in remote areas [3]. The investigation of distribution and density of these host animals are important in plague monitoring but are time and labor consuming. The results of early studies focused on the great gerbil [6] and marmot [7, 8], and the present research confirmed that remote sensing data with GIS methods are a good choice to analyze and predict the distribution and density of the host animals of plague.

### Ethical statement

In research of present paper, there is no sample of human and animal was included, therefore the ethical approval is not necessary for the research.

## Abbreviations

ASTER: advanced spaceborne thermal emission and reflection radiometer
ENM: ecological niche model
GDEM: global digital elevation model
GIS: geographical information system
GPS: global positioning system
LPDAA: Land Processes Distributed Active Archive Center
LST: land surface temperature
MODIS: moderate resolution imaging spectroradiometer
NASA: National Aeronautics and Space Administration
NDVI: normalized difference vegetation index
RS: remote sensing
